# TIMELAPSE study—efficacy of low-dose amitriptyline versus cognitive behavioral therapy for chronic insomnia in patients with medical comorbidity: study protocol of a randomized controlled multicenter non-inferiority trial

**DOI:** 10.1186/s13063-021-05868-4

**Published:** 2021-12-11

**Authors:** Nynke L. Rauwerda, Hans Knoop, Irene Pot, Annemieke van Straten, Marian E. Rikkert, Anouk Zondervan, Thom P. J. Timmerhuis, Annemarie M. J. Braamse, H. Myrthe Boss

**Affiliations:** 1 Department of Medical Psychology, Hospital Gelderse Vallei, Ede, The Netherlands; 2 Department of Medical Psychology, Amsterdam University Medical Centers, University of Amsterdam, Amsterdam Public Health Research Institute, Amsterdam, The Netherlands; 3 Department of Clinical Psychology & Amsterdam Public Health Research Institute, VU University, Amsterdam, The Netherlands; 4 Department of Medical Psychology, Hospital Rivierenland, Tiel, The Netherlands; 5 Department of Medical Psychology, Zaans Medical Center, Zaandam, The Netherlands; 6 Department of Neurology, Jeroen Bosch Ziekenhuis, ‘s-Hertogenbosch, the Netherlands; 7 Department of Neurology, Hospital Gelderse Vallei, Ede, The Netherlands

**Keywords:** Insomnia, Medical comorbidity, Amitriptyline, Cognitive behavioral therapy, CBT-I, Fatigue, Pain, Treatment preference, Non-inferiority

## Abstract

**Background:**

Insomnia is common in people with long-term medical conditions and is related to increased mortality and morbidity. Cognitive behavioral therapy for insomnia (CBT-I) is first choice treatment and effective for people with insomnia and comorbid long-term medical conditions. However, CBT-I has some limitations as it might not always be available or appeal to patients with medical conditions. Furthermore, a small proportion of patients do not respond to CBT-I. Preliminary evidence and clinical experience suggest that low-dose amitriptyline (AM) might be an effective alternative to treat insomnia in patients with medical comorbidity. In this randomized controlled trial, we will determine whether AM is non-inferior to the first choice treatment for insomnia, CBT-I.

**Methods/design:**

This study will test if treatment with low-dose amitriptyline for insomnia in patients with medical comorbidity is non-inferior to CBT-I in a multicenter randomized controlled non-inferiority trial. Participants will be 190 adults with a long-term medical condition and insomnia. Participants will be randomly allocated to one of two intervention arms: 12 weeks AM (starting with 10 mg per day, and if ineffective at 3 weeks, doubling this dose) or 12 weeks of CBT-I consisting of 6 weekly sessions and a follow-up session 6 weeks later. The primary outcome is subjective insomnia severity, measured with the Insomnia Severity Index (ISI). The primary endpoint is at 12 weeks. Secondary outcomes include sleep quality (e.g., sleep efficiency), questionnaires on daytime functioning (physical functioning and impairment of functioning), and symptoms (e.g., fatigue, pain, anxiety) at 12 weeks and 12 months post treatment and relapse of insomnia until 12 months after treatment.

**Discussion:**

Irrespective of the outcome, this study will be a much-needed contribution to evidence based clinical guidelines on the treatment of insomnia in patients with medical comorbidity.

**Trial registration:**

Dutch Trial Register NTR NL7971. Registered on 18 August 2019

**Supplementary Information:**

The online version contains supplementary material available at 10.1186/s13063-021-05868-4.

## Background

Insomnia disorder is prevalent (up to 66%) in patients with long-term medical conditions [1, 2]. Patients with a medical condition are 2.2 times more likely to have insomnia than patients without medical problems [3]. In DSM-5, insomnia disorder is defined as the complaint of poor sleep occurring at least 3 nights per week for at least 3 months with associated significant daytime effects [4]. Insomnia has been associated with an increased risk of various medical [5–7] and psychiatric disorders [8, 9] and injuries [10]. Sleep disorders in patients with medical conditions are assumed to negatively affect prognosis [11]. In patients with chronic illness, insomnia is independently associated with a significant decrease in overall quality of life [12]. The economic burden of insomnia in general is reported to be high. Health care use is reported to be twice as high among people with insomnia compared to those without, and insomnia-related work absence and reduced productivity are the main drivers of societal costs [13, 14]. Therefore, treatment of insomnia in patients with medical comorbidity is of utmost importance for health and functioning.

Insomnia treatments can be broadly divided into pharmacological (mainly benzodiazepine receptor agonists) and non-pharmacological. The European guideline for the diagnosis and treatment of insomnia recommends non-pharmacological treatment as first choice, of which cognitive behavioral therapy (CBT-I) is the most important one [15].

CBT-I is a multicomponent treatment package, applied face to face or online [15]. It includes behavioral and cognitive components, such as sleep hygiene education, stimulus control and sleep restriction interventions, relaxation techniques, and cognitive therapy. CBT-I has been found to improve sleep in about 70–80% of the participants both in individual and group treatment [16–19] and sustained effects were found on sleep and depression parameters [20]. A variety of studies have assessed the efficacy of CBT-I for insomnia comorbid with psychiatric and medical illness. Remarkably, the pre to post-treatment effect sizes in patients with comorbidities are comparable with those for primary insomnia and in some cases larger [21]. Furthermore, there is growing evidence that improved sleep following CBT-I positively influences the clinical course of the comorbid condition [22, 23].

In the short term, benzodiazepine receptor agonists (BZRAs) are as efficacious as CBT-I. In the long term, there is more support for efficacy of CBT-I in comparison to BZRA’s [24], given the higher relapse rates in BZRA’s when active treatment is discontinued. Therefore, the European guideline for the diagnosis and treatment of insomnia advises the use of BZRA’s only in short-term if CBT-I is ineffective or unavailable, in order to avoid their serious disadvantages, i.e., the rapid development of tolerance, dependence, adverse side effects and rebound effects [15]. Preventing adverse side effects (e.g., fall risk) is particularly relevant for patients with long-term medical conditions.

Despite this guideline, in the Netherlands BZRA’s are still often prescribed [25]. Several studies describe that clinicians often assume that patients with chronic insomnia prefer pharmacological treatment above psychological treatment [26–30]. Furthermore, CBT-I has several limitations. First, not all insomnia patients can access CBT-I, since CBT-I is still not widely implemented [28, 31] and conducting CBT-I in patients with medical comorbidity requires specialized practitioners [31, 32]. Second, not all insomnia patients are willing to undergo CBT-I. Stimulus control and sleep restriction are important components of CBT-I which mediate the reduction of insomnia, but initially, these components result in sleep deprivation and an increase of daytime sleepiness. The delayed therapeutic effect requires substantial motivation and effort. Patients with insomnia often perceive sleep restriction as unappealing and difficult to implement [33]. Adherence to stimulus control and sleep restriction has found to be relatively low, which negatively impacts outcome. In particular, patients with medical comorbidity can experience these components as strenuous or might be unable to fully engage in CBT-I due to their medical condition with associated disease burden. Third, there remains a proportion of individuals in which insomnia does not respond to (approximately 20%) or remits (40%) after CBT-I treatment [20]. Therefore, in addition to CBT-I as treatment for chronic insomnia, there is a need for effective, safe, and less strenuous alternatives for CBT-I that are suitable for patients with insomnia and long-term medical conditions.

In primary and secondary care settings in the Netherlands, several drugs are used off-label to treat insomnia [34], among which is amitriptyline in low dosage (AM) [35]. AM is a generic antidepressant commonly used in treatment of major depression. In low dosages, AM is also indicated for the treatment of neuropathic pain. Research to explore hypnotic efficacy in insomnia has not been done before [36], but clinical experience indicates that amitriptyline is effective in treating insomnia. Romenets e.a [37]. compared CBT-I with 6 weeks doxepin (a sedating tricyclic antidepressant as well) 10 mg at bedtime in patients with Parkinson’s disease and comorbid insomnia and found comparable effects.

Off-label prescription in general increases the risk of unnecessarily exposing patients to drugs without knowing how effective they are [38], which is undesirable, especially in a relatively fragile population with medical conditions. A head-to-head comparison of the current off-label use of AM as sleep medication and CBT-I is needed in patients with chronic insomnia and medical comorbidity. The randomized controlled non-inferiority TIMELAPSE trial will compare the use of low-dose AM with first choice of treatment (CBT-I) and determine if AM is non-inferior to CBT-I. Additionally, we want to explore mediators of the two treatments to improve knowledge of the working mechanisms leading to therapeutic change. Evidence suggests that CBT-I leads to changes in the cognitive, behavioral, and hyperarousal precipitating factors of insomnia (pre sleep arousal and dysfunctional attitudes and beliefs about sleep) [39]. Unclear is how AM will affect these elements. Finally, we wish to explore moderators of the treatment response, i.e., treatment preference, causal attributions of insomnia and insomnia subtypes [40].

## Objectives

### Primary objective

Our primary objective is to assess whether a 12-week treatment period of low-dose AM (10–20 mg nightly) is non-inferior with respect to its effect on insomnia severity compared to CBT-I in insomnia patients with long-term medical conditions.

### Secondary objective

Secondary objectives include to evaluate 1) relapse in insomnia rates in both groups up to 12 months post treatment, 2) secondary outcomes after 12 weeks and 12 months of treatment (sleep quality, fatigue, pain, depression, anxiety, physical functioning, impairment of functioning), 3) treatment evaluation (side-effects, adherence and withdrawal), and 4) possible mediators and moderators of treatment outcome.

## Methods/design

**Fig. 1:**
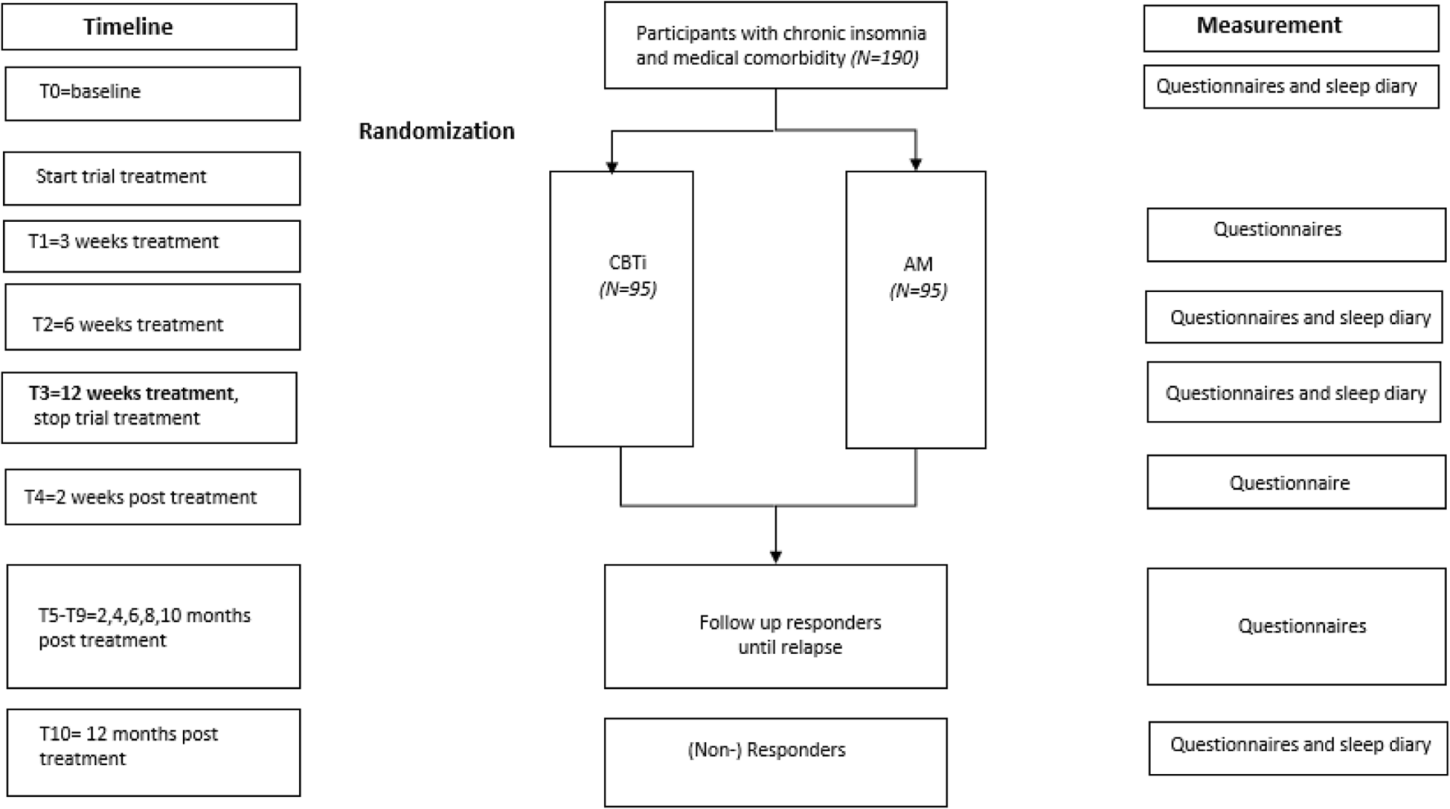
Trial flow chart

This study is a multicenter randomized controlled non-inferiority trial in which treatment with low-dose (10–20 mg nightly) AM is compared with the golden standard, CBT-I. Eligible consenting patients are randomized to 1) CBT-I or 2) low dose of AM, both for a period of 12 weeks in a ratio of 1:1. Patients are followed up to 12 months post treatment (thus 15 months post randomization). The study is implemented in regular care of the outpatient clinic of the departments of neurology (sleep-wake center) and departments of medical psychology of the participating Dutch hospitals (see Fig. 1 for a flow chart).

### Participants

Patients known with a long-term medical condition, aged 18–85 years old, suffering from insomnia disorder meeting DSM-5 criteria, and scoring ≥ 10 on the Insomnia Severity Index (ISI) [41] are eligible for participation [42]. A long-term chronic medical condition refers to all long-term medical conditions and/or persistent physical complaints (> 3 months) that require medical attention (in the form of consultation in medical health care, medication, aid or treatment) that cause dysfunction, discomfort, or social problems. The upper age limit is conform the guideline for low-dose amitriptyline treatment of neuropathic pain and the Food and Drug Administration approved use of doxepin (a sedating tricyclic antidepressant as well) in the treatment of insomnia. The DSM-5 criteria for insomnia disorder are sleep problems in at least 3 nights a week, for at least 3 months with consequences for daytime functioning, and the sleep problem cannot be better explained by or occurs exclusively during the course of another sleep disorder.

### Exclusion criteria

The study-related exclusion criteria are 1) habitual night shift worker, 2) an untreated sleep-related breathing disorder, 3) a wish to continue over-the-counter sleep aids as melatonin and medicinal cannabis, 4) the use of off-label amitriptyline for insomnia in the past year, 5) being unable to follow study instructions and fill out the study questionnaires in Dutch, 6) a known diagnosis of dementia or history of delirium and epilepsy, 7) pregnancy, lactation, or wish to become pregnant in the coming 6 months, 8) the presence of a severe psychiatric disorder (not in remission or not adequately treated), 9) current alcohol or drug abuse/addiction (benzodiazepine excluded), 10) terminal illness (life expectancy less than one year), 11) ocular hypertension/glaucoma, and 11) participation in other interventional studies. Furthermore, to prevent potential drug to drug interaction, subjects with 13) a current use of psychopharmaceuticals other than benzodiazepine such as antidepressants (selective serotonin reuptake inhibitors , tricyclic antidepressants, MAO-inhibitors), St John’s worth, anticonvulsants (e.g., carbamazepine), and antimyotics are excluded. Finally, subjects who are known with 14) any of the following contra-indications for amitriptyline conform pharmacological guidelines as used in clinical practice will be excluded: an allergy for amitriptyline, cardiovascular diseases (cardiac arrhythmia/blockade/long QT syndrome/Brugada syndrome, family history of acute cardiac death, recent myocardial infarction (within the past 90 days), and angina pectoris/coronary insufficiency), severe renal insufficiency (GFR < 10) or severe liver dysfunction. The exclusion criteria are based on guidelines of higher dosage amitriptyline. They are lined up to prevent, e.g., the risk of cardiac dysrhythmias, withdrawal symptoms in the newborn, and suicidal risk. Patients with dementia, severe psychiatric disorders (including suicidal risk), and patients who are not able to follow study instructions are excluded for the study, to prevent the risk of overdoses.

Table 1 summarizes the inclusion and exclusion criteria.

**Table 1:** Inclusion and exclusion criteria

Inclusion criteria	Exclusion criteria
18–85 years Insomnia disorder conform DSM-5 Score of ≥ 10 on the Insomnia Severity Index Long term medical condition	*Study-related exclusion criteria:* Habitual night shifts Untreated sleep-related breathing disorder Wish to continue over-the-counter sleep aids Off-label amitriptyline for insomnia in past year Epilepsy, dementia, history of delirium Pregnancy, lactation or pregnancy wish Terminal illness Ocular hypertension/glaucoma Severe psychiatric disorder Substance abuse/addiction (benzodiazepine excluded)
	*Potential drug-drug interactions for amitriptyline:* Use of psychopharmaceuticals (other than benzodiazepine) or antimyotics
	*Contra-indications for amitriptyline:* Allergy for amitriptyline Cardiac disorders Severe renal insufficiency or liver dysfunction

### Recruitment

Patients treated in medical health care (e.g., hospital, rehabilitation clinic, general practice) for their medical problems will be approached; treating physicians, nurse specialists, or nurse practitioners will inform patients who have complaints of insomnia with verbal and written information about the study during regular medical follow-up consults. Patients will also be informed about the study by leaflets and notifications on social media, narrow casting in the participating general hospitals, newsletters, and websites of patient associations.

If a patient is interested in the study, the general practitioner or medical specialist refers to the multidisciplinary diagnostic procedure for patients with long-term medical conditions and complaints of insomnia of the three participating general hospitals. In case patients are informed outside the care provision (e.g., by social media) and wants to participate (self-referral), a referral by a physician is required as well.

### Procedures

The multidisciplinary inclusion procedure consists of a consultation with a psychologist and a neurologist. During these appointments, criteria for insomnia (conform the DSM-5), (co)existence of other sleep wake disorders and possible exclusion criteria for treatment options are checked based on the referral information of the medical specialist or general practitioner (e.g., nature, severity and treatment of medical or psychiatric problem), medical history, clinical interview (e.g., medication use), and if necessary polysomnography (e.g., in case of complaints typically for sleep-related breathing disorder).

When a patient is considered eligible for the present study, the specialists involved in the procedure give brief information (orally) about the study, answer questions, and hand out the written patient information sheet and informed consent. Eligible patients will be informed (in a face-to-face contact or by phone) with a research assistant who answers questions, obtains the informed consent, and explains the study procedure.

Upon written consent, the baseline questionnaires and baseline sleep diary with instructions are provided online and the researcher or research assistant documents any over the counter medication. Finally, a double check on pharmacological eligibility is done by pharmacy of the participating hospital.

### Randomization and blinding

When the baseline questionnaires and the sleep diary are completed, the participants are randomized. Randomization is computer generated, by the webbased system (CastorEdc). Block randomization (blocks of 2 or 4) will be used to create similar distributions in the different study arms. Randomization into the two treatment groups is at patient level and will be stratified by benzodiazepine use, at least once every 2 weeks (i.e., no *versus* benzodiazepine use) at baseline and by referral type (self-referral versus referral by health care worker). Randomization will be executed centrally by a researcher/assistant who is not involved in the recruitment and inclusion of patients.

The research assistant informs the participants by phone about the result of the randomization procedure and explains the CBT-I appointment or how to collect the medication at the hospital pharmacy. The coordinating researcher and participant agree upon and document the date on which the participant will start taking/attending the study treatment.

It is not possible to blind participants or therapists to allocation; statistical analysis will be conducted by an independent statistician who will be blinded for group allocation.

## Interventions

### Medication

The treatment regime for the low dose of AM mimics current off-label practice as much as possible, i.e., a 12-week period of 10 mg AM, with the possibility of doubling the dosages by patients themselves at approximately 3 weeks, and medication stop at 12 weeks. Furthermore, participants who opt for a double dosage regime are allowed to return to a single dosage regime within the 12 week treatment. The participants in the AM group have a consultation with a neurologist at least once during treatment at approximately 6 weeks and at 12 weeks to evaluate the effect, dosage, and side-effects of treatment and guide medication stop. At the 6 weeks consultation, participants who doubled their dose to 20 mg at 3 weeks and wish to continue this double dose up to 12 weeks will receive prescription for a second badge AM as one badge AM is only sufficient for a single dose for 12 weeks. The neurologist inquires and motivates the adherence to medication during the appointment at 6 weeks after start of treatment. At the 12 weeks consultation, the neurologists motivate and guide medication stop.

### CBT-I

CBT-I consists of 6 weekly group sessions and a follow-up session at 12 weeks with a psychologist, specialized in insomnia and medical psychology. The treatment protocol is the manual of Verbeek et al. [43], and treatment elements are as follows: psycho-education, sleep hygiene, relaxation, behavior techniques such as stimulus control and sleep restriction, cognitive interventions, and relapse prevention. The degree of application of the behavioral techniques and sleep restriction is adapted to the medical condition (e.g., caution is advised with balance problems). If due to measures, for instance in the context of the COVID-19 pandemic, the group treatment cannot be offered face to face, the treatment will be offered by videoconferencing in group or individually via a secured connection. The psychologist inquires and stimulates adherence to CBT-I every session.

### Concomitant care during the trial

Subjects in the AM treatment group are advised to refrain from activities that require attention such as car driving when using AM in the first week. The use of other medication including benzodiazepines is allowed during participation in the trial. The only exception is the use of other psychopharmaceuticals. The general practitioner and the pharmacy of the patient is informed about study participation so that new prescriptions are checked for potential drug-drug interactions. Patients are instructed to refrain from using over-the-counter treatments as much as possible. We will monitor the use of both prescribed and over-the-counter sleep medication by questionnaire.

During the treatment period, all patients have access to written or online sleep hygiene advices provided by the participating hospital. In case of acute worsening of the insomnia due to a life-event or a comparable situation, other treatments might be prescribed by a medical doctor (such as benzodiazepines). This use will be monitored by self-report from the patient. Participants are instructed to report new or an increase of existing symptoms during the study to the clinician or researcher.

Adverse events will be treated and followed by the medical specialist conform usual practice. Participants who do not respond or relapse during the study or who request to end study participation will be invited to an individual follow-up session and offered further treatment outside the context of the study if required. The investigator and the treating physician in consultation with the research team can decide to withdraw a participant from the study for urgent medical reasons which are not predefined. The treatment can also be stopped at request of the patient. Those patients who choose to discontinue the treatment are offered the other treatment outside the context of the study.

## Assessments and outcomes

### Timing of assessments

Participants will be recruited from September 2019 to December 2022. They will be assessed at baseline (pre-randomization), 3, 6, and 12 weeks (end of treatment). Follow-up assessments will take place at 2 weeks, and at 2, 4, 6, 8, 10, and 12 months post treatment.

### Outcome measures

Primary outcome is insomnia severity measured with the Insomnia Severity Index (ISI) [41] after 12 weeks of treatment. The ISI is a 7-item questionnaire scored on a 5-point Likert scale reflecting the severity of both nighttime and daytime aspects of insomnia disorder as perceived by the participant in the last 2 weeks. Total scores range from 0 (no insomnia) to 28 (severe insomnia). A score of ≥ 10 is used to define clinical insomnia [44] .

We will use the Consensus sleep diary [45] for 1 week. Several variables will be calculated from the sleep diary: sleep efficiency (SE; percentage of time slept from the total amount of time spent in bed), sleep onset latency (SOL; time it takes to first fall asleep), number of awakenings (NOA), and total sleep time (TST, total number of hours the participant has slept). All these estimates are registered for 7 nights and for each variable a mean score over 7 days will be calculated.

Fatigue will be assessed with the subscale Fatigue severity of the Checklist Individual Strength (CIS-20), indicating the level of fatigue in the previous 2 weeks, measured with 8 items on a seven-point scale (range 8–56). A score of 35 or higher on the subscale indicates severe fatigue [46].

Anxiety and depression will be assessed with the Hospital Anxiety and Depression Scale (HADS) [47, 48], which has been extensively used in patients with medical conditions. The HADS contains 14 items (7 on depression and 7 on anxiety). A total score (range of 0–21) for each subscale is computed.

Pain severity and impact of pain on functioning will be measured with the 2-item subscale Bodily pain of the Short-Form 36-item Health Survey (SF-36) [49]. A total score is calculated ranging from 0 to 100. Higher scores indicate less pain and less impact of pain on functioning [50].

Impairment of functioning is assessed with the Work and Social Adjustment Scale (WSAS) [51]. The WSAS assesses five domains of functioning (work, home management, social leisure activities, private leisure activities, close relationships) with one item each. Each item is scored on an 8-point Likert scale. The usual timeframe (1 year) is adjusted to the past 2 weeks.

### Treatment evaluation

Adherence to CBT-I is assessed by a 5 item self-constructed questionnaire. This questionnaire assesses on a 5-point scale the extent to which participants followed each of their 6 treatment instructions (e.g., sleep hygiene advices, relaxation, use of sleep diary, sleep restriction and/or stimulus control, worry program and thought diary) since their prior visit. Item scores are added and the range of the total score is between 5 and 25. A higher score represents a better adherence. Furthermore, the number of missed sessions per participant will be tracked.

Treatment adherence to medication will be measured by pill count and a questionnaire (the Medication Adherence Rating Scale (MARS-5) [50] at 12 weeks. The MARS-5, a self-report instrument, contains 5 items regarding medication adherence. Each item is rated on a 5-point Likert scale, and the range of the total score is between 5 and 25. A higher score represents better medication adherence. Adherence by pill count will be calculated by the number of tablets provided minus the number of tablets returned to the investigator or reported as lost divided by the number of days on the study (correcting for dose) multiplied by 100.

Side-effects (including daytime sleepiness) are measured with the Antidepressant Side-Effect Checklist (ASEC) [52], which consists of 21 symptom items scored on a 4-point scale. An additional item asks whether the patient believes this symptom is linked to the treatment. Room for comments and additional side-effects is provided and three additional symptoms are asked. For this study, we split symptom 2 “Drowsiness” into “difficulty waking up/drowsiness when waking up” and “drowsiness during the day.” Symptom 3 “Insomnia” (difficulty sleeping) was split in “vivid dreams” and “disturbed sleep” as these might be side-effects of the treatment. The number, type, and severity of symptoms and their assumed link to treatment will be used to describe the experienced side effects. Daytime sleepiness (as a symptom) is measured by the Epworth Sleepiness Scale (ESS) [53]. The ESS is a 8-item questionnaire (scores 0–24) that requires participants to indicate how likely they would be to fall asleep or doze in various situations. ESS scores > 10 are considered to indicate excessive levels of daytime sleepiness.

For the AM group, withdrawal from medication will be measured with the 43-item Discontinuation-Emergent Signs and Symptoms (DESS) checklist [54], adjusted to self-administration format. Participants will be asked whether they have newly experienced one of the listed signs or symptoms during the first week after they have discontinued the treatment. The number of newly occurring events will be used to measure the effect of discontinuation.

### Moderators

The Treatment Perception and Preferences (TPP) measure [55] will be used to assess treatment preference and attributes that patients take into consideration when formulating their preferences. Nine items assess the treatment attributes related to each treatment (appropriateness, effectiveness, risks, convenience), which are rated on a 5-point scale (range 0–4). The item measuring risks will be recoded so that higher ratings on all items reflected a favorable perception of the treatment. The items are followed by two questions assessing treatment preference.

Type of insomnia will be assessed with two components of the Insomnia Type Questionnaire (ITQ) [40], namely the Subjective Happiness Scale (SHS) and the Positive And Negative Affect Scale (PANAS). The SHS [56] is a 4-item questionnaire to asses subjective happiness rated on a 7 point scale (range 1–7). Higher scores reflect greater happiness. The PANAS [57] contains 20 items on two subscales that assess a person’s positive and negative trait affect using a 5-point scale (1 = “very slightly or not at all”; 5=” extremely”).

Causal attributions which patients use to explain their insomnia will be assessed using 12 items of the Causal Attributions of my Insomnia questionnaire (CAM-I), developed by Harvey et al. [58]. The items reflect 12 domains that are commonly attributed to contribute to insomnia, such as sleep-related thoughts, bodily arousal, and lifestyle-related factors. Patients are asked the following question: “How likely do you think it is that the following factors contributed to your insomnia?” The domains are rated on a 7-point Likert scale. In this study, 3 domains (medical illness, bodily sensations and side-effects of pharmacological treatment for medical illness) will be added. At the end of the scale, an open-ended question will provide the possibility to list attributions that were not addressed by the modified CAM-I scale.

### Mediators

Dysfunctional beliefs about insomnia will be assessed with the 16-item brief Dysfunctional Belief and Attitudes about Sleep scale (DBAS-16) [59]. The sum of the DBAS score will be averaged so that the total score ranges from 0 (no dysfunctional beliefs) to 10 (severe dysfunctional beliefs).

Sleep-related arousal will be measured with the Pre-Sleep Arousal Scale (PSAS) [60]. The PSAS consists of 16 items that range from 1 (“not at all”) to 5 (“extremely”), higher scores indicating more arousal. Originally, the PSAS consists of two 8-item subscales concerning either cognitive or somatic arousal (*α* = 0.76–0.81). In correspondence with Vincent and Walsh [59], we report in this study on the sum-score of the PSAS.

All of the above questionnaires are freely available or permission for use has been granted by the authors.

### Additional measures

Current and previous use of sleep medication will be assessed by the neurologist-somnologist and registered in the electronic patient file/record. In addition to the self-report questionnaires, we will gather data on consultations (frequency of and reason for consultation, diagnoses on somatic and mental health) and prescription of medication from the electronic patient file during the year before and the year following the randomization.

Patient characteristics (i.e., sex, age, BMI, marital status, highest attained educational level, current work status, smoking status, alcohol consumption, duration of insomnia), life events in the past year, and additional means to improve sleep (e.g., vitamin preparation) are assessed in a self-constructed questionnaire.

Data will be collected and stored digitally using a structural database (CastorEDC). This database takes safety measures for collecting, storing, and processing data to ensure strict confidence and avoid disclosure of data to any third party without permission of the user. This system allows no missing items and sends reminders automatically. In case participants prefer a paper version of the questionnaires, this will be sent by regular mail and the filled in forms will be stored safely.

**Fig. 2:**
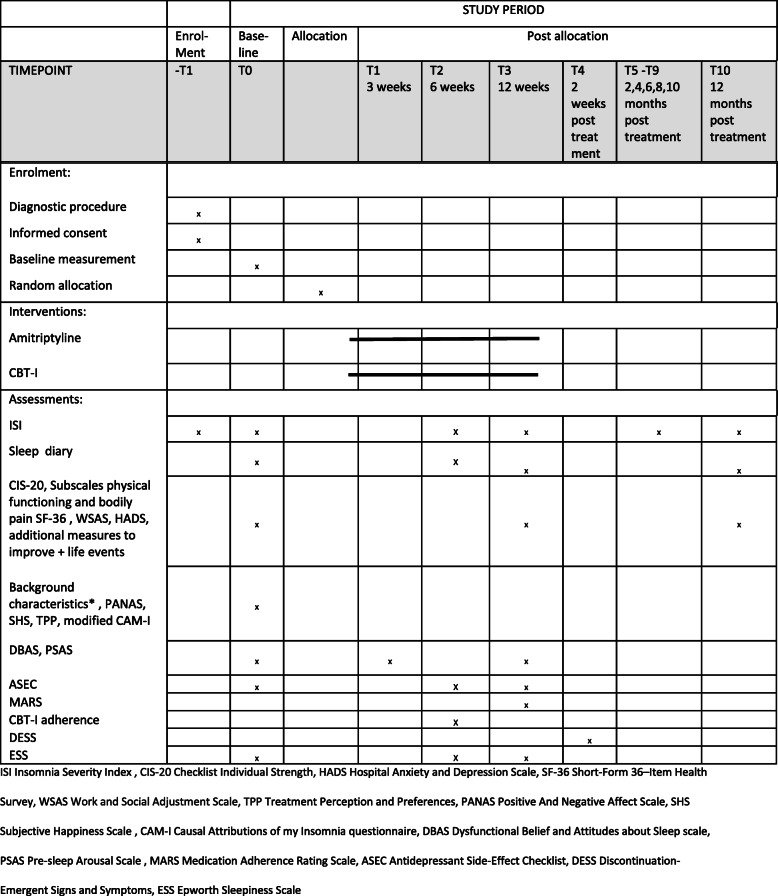
SPIRIT diagram

A diagrammatic representation of the trial process (enrolment, intervention and assessment) is shown in the Standard Protocol Items: Recommendations for Interventions (SPIRIT) diagram (Fig. 2). For the full SPIRIT checklist, please see supplementary material Additional file 1.

### Sample size

We hypothesize that the effect of low-dose AM at 12 weeks is non-inferior to the golden standard CBT-I. A low-dose AM will be considered non-inferior to CBT if the mean post treatment ISI total score does not exceed that of the CBT-I group by more than 4 points, i.e., 50% of the minimum clinical significant difference reported following CBT-I. The standard deviation of the ISI in our study is assumed 4.4 [61]. Sample size determination following the recommendations outlined in Tamayo-Sarver et al. [62] using a one-tailed test and a 5% significance level showed that 84 participants in each group will provide adequate power (90%) to reject the null hypothesis that the ISI change produced by low-dose amitriptyline is not similar to those produced by CBT-I. The dropout rate is estimated to be 13% [41]. Hence, 190 participants in total will be included.

### Statistical analysis

Demographic and clinical characteristics (e.g., age, gender, medical condition, preference and type of insomnia) and primary and secondary outcomes at baseline (e.g., sleep efficiency, emotional complaints) will be presented in means (standard deviations) and numbers (percentages) depending on their distribution.

The null hypothesis states that there is a difference in mean scores on the primary outcome between the treatment groups; the alternative hypothesis is that the difference in mean scores on the primary outcome measure does not differ by more than the non-inferiority margin. The primary analysis will be conducted according to intention-to-treat, i.e., all randomized patients are included and are analyzed in their randomly assigned treatment group. Outcomes that are based on measurements at multiple time points, i.e., weeks 6 and 12, are analyzed using mixed linear models. Mixed linear models can handle the presence of missing data without the need for imputation and still provide valid estimates of treatment effects.

The primary outcome for analysis is the mean subjective insomnia severity as assessed using the ISI at week 12. To test for the difference between the two treatment groups, the primary outcome of ISI score at week 12 will be analyzed using analysis of covariance (ANCOVA) with treatment group (amitriptyline or CBT-I) as the independent variable and the baseline value as a covariate. We will check for violations of necessary assumptions of ANCOVA (normality, homogeneity of variance and random independent samples). We will calculate a one-sided 95% confidence interval around the mean difference in the primary outcome between the treatment groups to determine if the upper limit of the confidence interval will be below the non-inferiority margin of 4 points. Amitriptyline will be declared non-inferior to CBT-I if the upper bound of the 95% confidence interval does not exceed the non-inferiority margin of 4 points. If amitriptyline is shown to be non-inferior, we then will proceed to superiority hypothesis testing. For superiority testing, we will consider the two sided 95% confidence interval of the mean difference between both groups on the primary outcome as established in the ANCOVA and verify if it includes zero or not.

The primary analysis will be conducted according to intention-to-treat, i.e., all randomized patients are included and are analyzed in their randomly assigned treatment group. We will use multiple imputations to handle missing data for outcomes that are based on the measurement at a single time point, including the primary outcome of the ISI score at week 12. We will include all baseline demographic and clinical characteristics in the imputation model. We will impute a total of five datasets and these will be pooled according to Rubin’s rule [63].

Additionally, the mean ISI score over time will be compared between the two treatment groups using linear mixed models with treatment group (amitriptyline or CBT-I) and time (6 and 12 weeks from baseline) included as fixed effects and ISI score at baseline included as covariate.

We will repeat the ANCOVA and the linear mixed models analysis of the ISI score as a per protocol analysis in which only patients who actually received their assigned treatment (in the CBT-I group attendance of at least 5 out of 7 sessions and in the AM group at least 70 out of 84 days medication) will be included.

Percentages responders until relapse at 2, 4, 6, 8, 10, and 12 months post treatment in both treatment groups will be compared using survival analyses. We will express the difference between amitriptyline and CBT-I in effect sizes and percentage responders. Treatment response is defined as ≥ 8 point reduction on ISI. A relapse is defined as a drawback of ≤ 8 points to the baseline score of the Insomnia Severity Index measured during follow-up at two subsequent measurements.

ANCOVA will be repeated on the secondary outcome measures assessed at week 12 and 12 months, adjusted for the baseline measurement and the linear mixed models will be repeated for the secondary outcomes sleep quality, fatigue, emotional complaints, physical functioning, impairment of functioning, and pain, all assessed at weeks 6 and 12 and 12 months post treatment adjusted for the baseline measurement, according to intention to treat and per protocol analyses. Bonferroni corrections will be applied to the p-values to account for multiple comparisons.

Rates of reported side effects, withdrawal, and rebound symptoms as well as the treatment evaluation items will be described.

The demographic characteristics will be used to identify potential subgroups. Moderators (data on demographic variables, type of insomnia, treatment preference) will be investigated to see whether these factors influence the intervention’s effectiveness (power of the analyses permitting). We will explore the extent to which these variables moderate the effect of the interventions by including interaction terms between these variables on the one hand and intervention type on the other hand in the ANCOVAs.

Mediation analysis will be conducted to explore the possible underlying mechanisms of the level of reduction in subjective insomnia after amitriptyline or CBT-I at week 12. We will perform multiple mediation analysis using bootstrapping to test the mediating effect of the potential mediators (pre sleep arousal and dysfunctional cognitions and beliefs about sleep).

### Ethics and dissemination

The study has been approved by the Medical Ethics Committee of Amsterdam University Medical Centers, University of Amsterdam (2019_101), the competent authority Central Committee on Research Involving Human Subjects (Centrale Commissie Mensgebonden Onderzoek) and the ethics committees of the participating hospitals. All treatments are based on best evidence and expected to benefit participants. Because all treatments are well-known procedures and the trial cannot be blinded, no data management committee will be required. Detailed monitoring procedures will be described in a study-specific monitoring plan.

The results of research will be submitted for publication to peer-reviewed scientific journals and both positive and negative findings will be disclosed, unreservedly. There are no restrictions placed upon publication by the sponsor of this study (Hospital Gelderse Vallei, Ede, the Netherlands).

This study will be conducted according to the principles of the Declaration of Helsinki (Version October 2013, adopted at the 64th WMA General Assembly, Fortaleza, Brazil) and in accordance with the Medical Research Involving Human Subjects Act (WMO). Guideline for Good Clinical Practice (ICH-GCP); the Dutch Medicines Act and the Dutch data protection regulation.

Only the principal investigator and dedicated research assistants will be able to access the source data. Data will be kept for 15 years. After that, the data will be anonymized (keyfiles destroyed).

The data will be coded in such a way that they cannot directly be traced back to the identity of the participant. Only the project leader and primary researcher have access to the key file with codes and participant data. Codes and participant data will be stored in password-protected files. After finishing the study, the key to the code will be safeguarded by the coordinating investigator.

## Discussion

This study protocol, which adheres to SPIRIT 2013 [64], describes the first randomized controlled trial evaluating whether AM is non-inferior to first choice treatment for insomnia CBT-I in treating insomnia in patients with medical conditions. The results will offer valuable data about the efficacy of treatments for insomnia in patients with a chronic medical condition.

This study has some limitations. First, neither the practitioner nor the participants can be blinded to allocation. Second, patients with strong preferences for one of the treatments (e.g., AM or CBT-I) may be less willing to participate in the study as randomization can allocate them to the non-preferred treatment. Therefore, we will assess treatment preference and explore its effect on the primary and secondary outcomes. Furthermore, we will assess drop out after randomization. Third, due to the potential drug-drug interactions for amitriptyline, patients using psychopharmaceuticals (other than benzodiazepines) are excluded from the study. The use of psychopharmaceuticals can be related to more severe disease burden. This might result in an underrepresentation of patients with severe disease burden. Fourth, in the present study, we focus on the non-inferiority of both treatments. If shown to been non-inferior, future research should include a cost analysis to compare the economic impact of both treatments

The results of this trial will be of clinical relevance in the treatment of insomnia for patients with long term medical conditions. If the results do not show low-dose amitriptyline is as effective as CBT-I, CBT-I will remain first choice treatment especially in this relative fragile population with comorbid medical conditions. On the other hand, if the results indeed demonstrate that both interventions lead to a similar reduction of insomnia and comparable treatment evaluation, an effective, safe, and low-cost alternative pharmacological treatment will become available for chronic insomnia patients with a long term medical condition. The results can also provide insights in effects of both treatments on daytime symptoms, so patients and caregivers can be adequately informed in order to decide which treatment to choose. Furthermore, the pharmacological treatment option might prevent (new) insomnia patients becoming dependent on benzodiazepines. Research on mediators is valuable for improving knowledge of the working mechanisms of a treatment that will lead to therapeutic change. In turn, these elements can be intensified and refined, which can make treatment more effective and less costly. Identifying moderators of treatment outcome enables the tailoring of treatment options (psychological or pharmacological treatment) to individual patients.

## Trial status

Recruitment is ongoing. Recruitment began in September 2019 in Hospital Gelderse Vallei, Ede, the Netherlands. From November 2020, recruitment started in Hospital Zaans Medical Centre, Zaandam, and Hospital Rivierenland, Tiel, and from June 2021 in Hospital Jeroen Bosch, ‘s-Hertogenbosch, all in the Netherlands as well. At the time of submitting the protocol, 75 of 190 participants (39%) have been consented and 68 randomized. Current protocol version 4.3 is dated August 2020.

## Supplementary Information

**Additional file 1.** SPIRIT 2013 checklist: Recommended items to address in a clinical trial protocol and related documents.

## Abbreviations

AM: Amitriptyline
ANCOVA: Analysis of covariance
ASEC: Antidepressant side effect checklist
BZRA: Benzodiazepine receptor agonists
CAM-I: Causal attributions of my insomnia
CBT-I: Cognitive behavioral therapy for insomnia
CIS-20: Checklist individual strength
DBAS: Dysfunctional beliefs and attitude about sleep scale
DESS: Discontinuation-emergent signs and symptoms
DSM-5: Diagnostic and statistical manual of mental disorders 5
ESS: Epworth sleepiness scale
GCP: Good clinical practice
HADS: Hospital anxiety and depression scale
ISI: Insomnia severity index
ITQ: Insomnia type questionnaire
PANAS: Positive and negative affect scale
PSAS: Pre-sleep arousal scale
RCT: Randomized controlled trial
SHS: Subjective happiness scale
SPIRIT: Standard protocol items: recommendations for interventions
SF-36: Short-form 36–item health survey
TPP: Treatment perception and preferences
WSAS: Work and social adjustment scale
